# A Rare Penetrating Trauma of Both Orbit and Nasal Cavity

**Published:** 2018-11

**Authors:** Mohammad-Waheed El-Anwar

**Affiliations:** 1 *Department of Otorhinolaryngology, Head and Neck Surgery, Faculty of Medicine, Zagazig University, Zagazig, Egypt.*

**Keywords:** Foreign body, Nose, Orbit, Pencil, Septum

## Abstract

**Introduction:**

Diagnosis of orbital foreign body (FB) penetration is usually obvious when part of the FB is still attached at the entry wound (1). However, the depth and course of the FB in this case was not visible.

**Case Report:**

A 5-year old female presented with a pencil penetrating the left orbit. A computed tomography (CT) scan showed that the pencil penetrated the left orbit (extraseptal) through the lacrimal bone to the left nasal cavity, then perforated the nasal septum, crossing the right nasal cavity. Finally, the pencil penetrated the lamina paperatea to the right orbit and stopped near the right optic nerve. The pencil was gently removed under general anesthesia with close observation of the eyes.

**Conclusion:**

A case of a pencil penetrating both orbits and nasal cavities was reported, and the pencil was safely removed. This draws attention to the possible penetration power of a pencil, with the possibility of injury to the orbit and optic nerve on the opposite side of the penetration. It also demonstrates the feasibility of safe removal.

## Introduction

The orbit has pyramidal shape and is formed by thin bony walls. In children, the orbital walls are very thin and can be fractured by a low-velocity penetrating foreign body (FB). Thus, orbital penetrating injury is more common in children than in adults (1).Penetrating wounds to the orbit constitute 30–50% of all traumatic eye injuries (2). The orbital contents or globe, or both, may be severely damaged with resulting blindness in the affected eye (3). Diagnosis of FB penetration is usually obvious when part of the FB is still attached at the entry wound (1). However, the depth and course of this FB was not visible. We report the first case of an uncomplicated pencil FB penetrating both orbital cavities, both nasal cavities and the nasal septum, and describe its management.

## Case Report

**Fig 1:**
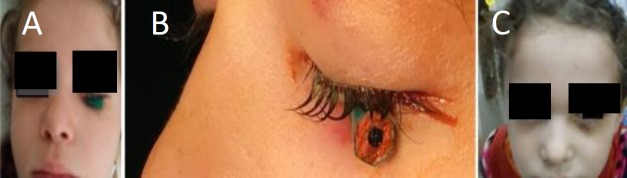
A: Penetrating pencil entering transversely; B: Close view of the pencil; C: 1-week post removal

A 5-year old female presented with a wooden FB (pencil) penetrating the left orbit after falling while the pencil was in her hand at home. On examination, part of the pencil appeared at the entry wound inferior and lateral to the medial canthus, with mild right upper lid edema (Fig.1). On ophthalmological examination, visual acuity and ocular mobility was not affected and both globes were intact. Both pupils were reactive.

**Fig 2:**
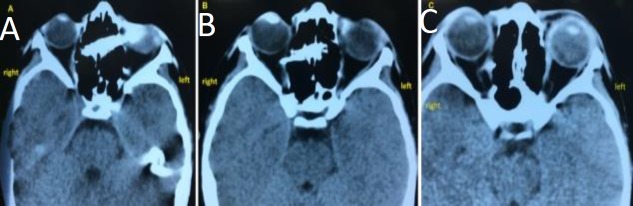
CT of the penetrating pencil showing A: the pencil penetrating the right orbit and both nasal cavities; B; Pencil penetrating to the left (contralateral) orbit; C: Tip of the pencil stopping close to the contralateral optic nerve.

A computed tomography (CT) scan showed that the pencil penetrated the left orbit through the lacrimal bone to the left nasal cavity then perforated the nasal septum, crossing the right nasal cavity. From there, it penetrated the lamina paperatea to the right orbit until its tip stopped very close to the right optic nerve (Fig. 2).

**Fig 3:**
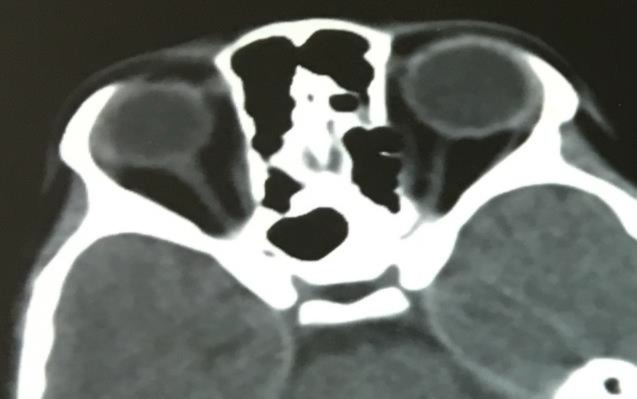
1-month postoperative CT without negative sequelae on both orbits and nasal cavities

The pencil was gently removed by slow gradual out traction under general anesthesia with close observation of the eyes. After removal, no epistaxis, cerebrospinal fluid (CSF) leak or orbital hematoma was detected. Repair of the entry wound was performed using 5-0 Vicryl in two layers. Postoperatively, visual acuity and ocular mobility were found to be normal, with both globes intact and both pupils reactive. No epiphora or nasal synechia or anosmia were observed. After pencil extraction, no significant destruction of the orbits or the nose was detected by history, examination or CT (Fig.3).

*Ethical standards*

The author obtained written informed consent from the parents of the reported case and the study was approved by the Institutional Review Board.

## Discussion

Penetrating orbital injury may lead to serious sequelae because vital structures could be compromised (3). Even when intracranial penetration is ruled out, the presence of optic nerve sheath hematoma, orbital hematomas, abscesses and some FBs (organic materials or copper, for example) are considered true emergencies (4). Standard pencils (containing graphite and dry wood) designed for writing have a conical-shaped end with a pointed tip that increases the feasibility and depth of penetration.

In the current report, part of an orbital penetrating pencil was apparent from the penetrating wound. The child sought medical advice on the same day of penetration, and therefore no infection was reported. This contrasts with the case reported by Al-Otaibi and Baeesa (5), in which the patient asked for medical advice after 3 days. Since the orbit has a close proximity to the paranasal sinuses, infection (particularly abscess formation) is a common antecedent from the penetrating orbital injury (3). Therefore, radiological imaging is indicated in cases with orbital penetrating wounds.

The course of the current FB penetration suggested that, even though the FB was non-metal and obvious, CT was mandatory before removal in order to evaluate the effect of its penetration not only on the orbit and nasal cavity but also on the opposite orbit that may also have been affected. Thus, CT is the imaging study of choice for such a penetrating orbital injury (6).

Our case was not associated with a complicated course, did not lead to any functional sequelae, and the visual acuity on presentation was not compromised. It was important to remove the pencil FB to avoid infection and complications, and this was done safely by gentle extraction in our case.

The current report is the first case of an uncomplicated penetrating pencil affecting both orbital cavities, both nasal cavities and the nasal septum. 

The FB was safely removed by gentle extraction without related sequelae. This case draws attention to the possible risk of penetration and injury of the opposite orbital structure by penetrating the wooden FB to one orbit. Thus, early diagnosis and defining of the course of penetration by CT is mandatory. Moreover, early intervention allows low-risk removal and obviates the risk of infection.

## Conclusion

A case of penetrating pencil of both the orbital cavities, both nasal cavities and the nasal septum was reported and safely extracted. This case draws attention to the risk of injury of the contralateral orbital structure by a penetrating wooden FB to one orbit, and the benefits of early diagnosis to allow easy and safe removal and obviate risk of infection.
